# Modifying the yeast very long chain fatty acid biosynthetic machinery by the expression of plant 3-ketoacyl CoA synthase isozymes

**DOI:** 10.1038/s41598-022-17080-8

**Published:** 2022-08-02

**Authors:** Kenna E. Stenback, Kayla S. Flyckt, Trang Hoang, Alexis A. Campbell, Basil J. Nikolau

**Affiliations:** 1 Roy J Carver Department of Biochemistry, Biophysics and Molecular Biology, Iowa State University, Ames, IA USA; 2 Center for Metabolic Biology, Iowa State University, Ames, IA USA; 3 Present Address: Department of Biological Chemistry and Molecular Pharmacology, Harvard Medical School Blavatnik Institute, Boston, MA USA; 4 Present Address: Corteva Agriscience, Johnston, IA USA; 5 Present Address: Department of Chemical Engineering, University of Michigan, Ann Arbor, MI USA; 6 Present Address: School of Education, Iowa State University, Ames, IA USA

**Keywords:** Biochemistry, Biological techniques, Cell biology, Chemical biology, Genetics, Molecular biology, Plant sciences

## Abstract

Eukaryotes express a multi-component fatty acid elongase to produce very long chain fatty acids (VLCFAs), which are building blocks of diverse lipids. Elongation is achieved by cyclical iteration of four reactions, the first of which generates a new carbon–carbon bond, elongating the acyl-chain. This reaction is catalyzed by either ELONGATION DEFECTIVE LIKE (ELO) or 3-ketoacyl-CoA synthase (KCS) enzymes. Whereas plants express both ELO and KCS enzymes, other eukaryotes express only ELOs. We explored the *Zea mays* KCS enzymatic redundancies by expressing each of the 26 isozymes in yeast strains that lacked endogenous ELO isozymes. Expression of the 26 maize KCS isozymes in wild-type, *scelo2* or *scelo3* single mutants did not affect VLCFA profiles. However, a complementation screen of each of the 26 KCS isozymes revealed five that were capable of complementing the synthetically lethal *scelo2; scelo3* double mutant. These rescued strains express novel VLCFA profiles reflecting the different catalytic capabilities of the KCS isozymes. These novel strains offer a platform to explore the relationship between VLCFA profiles and cellular physiology.

## Introduction

Very long chain fatty acids (VLCFAs) are defined as fatty acids with chain lengths that are greater than 18 carbon atoms; in some organisms they can be up to 50 carbon atoms and longer^1,2^. As building blocks for the assembly of more complex molecules, VLCFAs contribute to a variety of cellular functions, including energy storage (e.g., triacylglycerols), biochemical signaling (e.g., sphingolipids), and membrane structure (e.g., phosphoglycerolipids)^3,4^. In many organisms, including plants, insects, and mammals, a subset of VLCFAs are also the building blocks of an extracellular structure, the cuticle, which coats the exterior of these organisms, providing the primary physical barrier to the environment^5,6^. While VLCFAs account for a minor portion of the fatty acid pool within cells, their importance is illustrated by the fact that the absence of VLCFAs is lethal in fungi^7^, plants^8,9^ and animals^10–12^.

VLCFAs are generated by an ER-localized enzyme system, called fatty acid elongase (FAE)^13,14^ that utilizes preexisting fatty acids substrates, which are generated de novo from acetyl-CoA by a fatty acid synthase (FAS) system^15^. Both the de novo FAS and the FAE systems utilize cyclical iterations of Claisen condensation-reduction-dehydration-reduction reactions to elongate an acyl-chain by 2-carbon atoms per cycle. In contrast to the FAS system, which utilizes acyl carrier protein (ACP) bound acyl intermediates, the FAE system utilizes CoA-bound acyl intermediates in these reactions.

Two types of non-homologous enzymes catalyze the initial Claisen condensation reaction of FAE. One of these is the FAE1-like 3-ketoacyl-CoA synthases (KCS-type enzymes), and the other is the ELONGATION DEFECTIVE-LIKE (ELO)-type enzymes. FAE1 was initially identified in Arabidopsis, and these KCS-type enzymes are only found in plants. ELO-type enzymes were initially identified in the yeast, *Saccharomyces cerevisiae*^7,16^, and they are more widely distributed among eukaryotic organisms, including plants, fungi and animals^10,16,17^. The reaction catalyzed by both of these enzymes involves the condensation between an acyl-CoA and malonyl-CoA, generating a 3-ketoacyl-CoA product that is 2-carbon atoms longer than the initial acyl-CoA substrate. The subsequent three reactions of each FAE cycle are sequentially catalyzed by the 3-ketoacyl-CoA reductase (KCR), 3-hydroxyacyl-CoA dehydratase (HCD), and enoyl-CoA reductase (ECR) components, and cumulatively these reactions chemically reduces the 3-keto functional group to a methylene group, and the resulting acyl-CoA product is the substrate for the next Claisen condensation reaction of the FAE cycle.

The occurrence of both KCS- and ELO-type enzymes in plants provides biochemical redundancy in the generation of VLCFAs, which is even further enhanced by the genetic redundancy that occurs in the genomes of these organisms. For example, the Arabidopsis and *Zea mays* (maize) genomes appear to encode for 21 and 26 KCS isozymes, and four and six ELO isozymes, respectively^17^. This apparent redundancy of enzymes catalyzing the Claisen condensation reaction of FAE is thought to be universal among terrestrial plant species, suggesting an evolutionary advantage^18,19^.

In this study we explored the redundancy among the plant *KCS*-coding genes. We expressed each maize KCS isozyme in a wild-type yeast strain, or in yeast strains that functionally lacked either individually or in combination two of the three *ELO*-coding genes. Specifically, we evaluated the impact of expressing each of the 26 *Zm*KCS isozymes on the growth and fatty acid profiles of wild-type (WT), *scelo2* and *scelo3* single mutant strains, as well as the ability of these *Zm*KCS isozymes to genetically complement the synthetically lethal *scelo2; scelo3* double mutant strain.

## Results

### *ZmKCS* expression in the WT BY4742 background

Initial characterizations involved the individual expression of the 26 *ZmKCS* sequences in a yeast strain that contained an intact, WT FAE system. Expression of 11 of the *Zm*KCSs caused a statistically significant abatement of growth as compared to the recipient strain. Notably, five of these strains, expressing *Zm*KCS9, *Zm*KCS18, *Zm*KCS19, *Zm*KCS24, or *Zm*KCS27, showed a 25% increase in doubling times, and with the other six strains (*ZmKCS2, ZmKCS3, ZmKCS10, ZmKCS11, ZmKCS21,* and *ZmKCS23*), doubling time was increased by ~ 5–10% (see Supplementary Fig. S1a).

Fatty acid analyses of the individual strains expressing each of the *Zm*KCS isozymes identified fatty acids that normally accumulate in WT yeast strains^20^, ranging between 10-carbon and 26-carbon chain lengths, including monounsaturated fatty acids and fatty acids that are hydroxylated at the 2-position (see Supplementary Fig. S1c and e). These fatty acids were evaluated as products of either de novo fatty acid biosynthesis, a process catalyzed by the fatty acid synthase (FAS) complex (i.e., the FAS1/FAS2 complex), which generates fatty acids of up to 18-carbon chain length (see Supplementary Fig. S1b and c), or as products of the FAE system that generates the longer chain fatty acids of up to 26-carbon chain length (see Supplementary Fig. S1d and e)^21^. Among the 26 *Zm*KCS expressing strains, only the expression of *Zm*KCS2 and *Zm*KCS3, showed statistically significant quantitative or qualitative changes in the fatty acid profiles. Specifically, expression of *Zm*KCS2 increased the FAE-generated products, including statistically significant increases in 24 and 26-carbon fatty acids, whereas the expression of *Zm*KCS3 induced a quantitative increase (by 25%) of the products derived from FAS, without qualitatively affecting the fatty acid profiles that were produced by the recipient strain. The expression of all the other *Zm*KCS proteins had no significant quantitative effects, and only had subtle effect on the FAE- or FAS-derived product profiles (see Supplementary Table S3).

### *ZmKCS *expression in the yeast *scelo2* mutant background

All 26 *Zm*KCS isozymes were individually expressed in a yeast mutant strain that lacked a functional *scelo2* gene. The *scelo2* mutation did not affect the growth of the strain as compared to the strain that carried a WT yeast FAE. Moreover, adding each *Zm*KCS into this *scelo2* mutant strain also did not affect the growth of the strain. Indeed, except for the *Zm*KCS21-expressing strain, there was no significant change in growth rates of these strains as compared to the strain that carried the WT yeast FAE system (see Supplementary Fig. S2a).

Fatty acid analyses of these strains show that the most significant impact of the *scelo2* mutation is the hypo-accumulation of FAE-generated VLCFAs (i.e., 50% reduction as compared to the WT strain) (see Supplementary Fig. S2d). Most notably are the significant reductions in 20- and 26-carbon fatty acids (~ 85% decrease as compared to WT). Despite these changes in VLCFA accumulation, as expected the disruption of the *scelo2* gene had only minor statistically significant effects on the fatty acids produced by the FAS system; the most significant modulation being the increased accumulation of minor FAS constituents, including C15- and C17-carbon fatty acids, as well as their 2-hydroxy derivatives (five–tenfold increase, see Supplementary Table S3).

The individual expression of the 26 *ZmKCS* genes in this *scelo2* mutant background did not significantly change the VLCFA content as compared to the recipient strain and caused only subtle, insignificant changes in the relative abundances of individual VLCFAs (see Supplementary Fig. S2e). Furthermore, the expression of individual *ZmKCS* genes in this mutant background had minor or statistically insignificant changes in the FAS-derived products (see Supplementary Fig. S2b and c and Supplementary Table S3).

### *ZmKCS *expression in the yeast *scelo3* mutant background

The lack of a functional *scelo3* gene did not significantly affect the growth of the yeast strain (see Supplementary Fig. S3a). However, the expression of 11 specific *ZmKCS* genes in this mutant strain significantly reduced growth, increasing doubling time by 10–40%. These strains were also analyzed to assess their ability to produce fatty acids. As previously established^7^, the *scelo3* mutation caused a large (~ tenfold), quantitative increase in the accumulation of VLCFAs as compared to the WT control strain (see Supplementary Fig. S3d). Most notably, this was affected by the loss of the 26-carbon VLCFA and its hydroxylated derivative, and this is coupled with a reciprocal increase in the accumulation of 20-carbon, and 22-carbon VLCFAs and 2-hydroxy-derivatives (see Supplementary Fig. S3e). With the exception of the *Zm*KCS15-expressing strain, the expression of the other *ZmKCSs* in this *scelo3* mutant did not change this profile. On the other hand, the *Zm*KCS15-expressing strain was able to produce statistically significant increased quantity of 24-carbon VLCFAs, and detectable quantity of 26-carbon VLCFAs and their hydroxylated derivatives (see Supplementary Table S3).

Parallel evaluation of the FAS-generated fatty acids produced by these strains, indicates that the majority of the *Zm*KCS-expressing strains (21 strains) did not change the accumulation of these fatty acid products. The other five *Zm*KCS-expressing strains had minor, but statistically significant effects on the accumulation of these FAS-generated fatty acid products (see Supplementary Fig. S3b and c); *Zm*KCS2 and *Zm*KCS18 increased these fatty acids by ~ 15%, and the expression of *Zm*KCS24, *Zm*KCS25, and *Zm*KCS27 lead to ~ 30% decrease in FAS products. In the latter three strains, a significant reduction in 18:1 fatty acid was observed (50–70% reduction as compared to the *scelo3* mutant strain).

### Genetic complementation of the synthetically lethal *scelo2; scelo3* double mutant by *ZmKCS* expression

**Figure 1:**
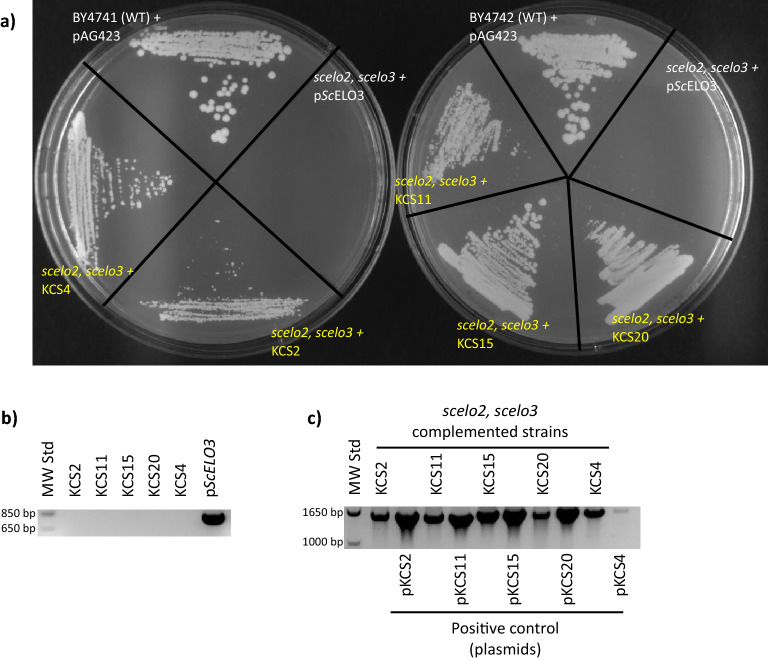
*ZmKCS*-expressing strains complement the lethality of the *scelo2; scelo3* double mutant. Out of the 26 *Zm*KCSs evaluated, the expression of five *Zm*KCS isozymes were able to restore the viability of the synthetically lethal phenotype of the *scelo2; scelo3* double mutant strain. (**a**) Growth of the indicated strains on SD his- media containing 5-FOA; controls include the WT BY4741 and BY4742 strains harboring the empty pAG423-GPD vector, and the *scelo2; scelo3* double mutant strain whose viability was maintained by the ectopic expression of *ScELO3*. (**b**) PCR molecular confirmation of the absence of the ectopic *ScELO3* gene in the recovered *Zm*KCS complemented strains. Template DNA was amplified with the *ScELO3*-specific primers (5′-GCCGACCAGTTCCAGTCTTT and 5′-CCGTCCAAGTATTTGTGAGC). (**c**) PCR molecular confirmation for the presence of the *ZmKCS* sequence in the recovered *ZmKCS* complemented strains. Primers for this assay are listed in Table S1.

The ability of the 26 *ZmKCS* genes to complement the synthetic lethality of the *scelo2; scelo3* double-mutant was determined by a plasmid-shuffle strategy. In these experiments we expressed each *Zm*KCS isozyme in a strain that carried an episomal wild-type *ScELO3* allele, which maintained the viability of the otherwise synthetically lethal *scelo2; scelo3* double-mutant strain. Because the episomal wild-type *ScELO3* allele also carried the *URA3* selection marker, growing these strains on 5-FOA-containing media counter-selected the episomal *ScELO3* allele, and strains that expressed a *ZmKCS* capable of complementing the *scelo2; scelo3* double mutant lethality were recovered. These experiments resulted in the recovery of five *ZmKCS* genes (*ZmKCS2, ZmKCS4*, *ZmKCS11*, *ZmKCS15,* and *ZmKCS20*), demonstrating their ability to complement the lethality associated with the *scelo2; scelo3* double mutant (see Fig. 1).

**Figure 2:**
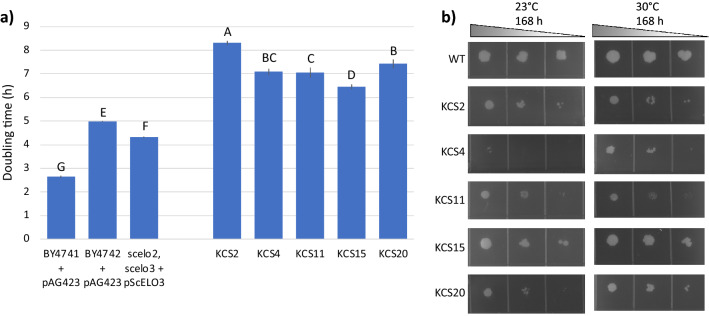
Growth of the *ZmKCS* complementing *s*trains*.* (**a**) Culture doubling time of the *scelo2; scelo3* double mutant strain complemented by the expression of *Zm*KCS isozymes. Error bars represent standard error from 3 replicates, different letters above the data-bars indicate statically significant differences based on Student’s t-test (*p* < 0.05). (**b**) Serial tenfold dilution inoculum of each indicated strain was applied on solid media, and growth was evaluated at 23 °C and 30 °C at the indicated times after inoculation. Images are representative from three repetitions of this experiment.

Even though the recovered strains were viable, they each suffered a significant growth penalty as compared to the WT strain, increasing doubling time by between two- and four-fold as compared to the BY4741 and BY4742 WT strains, and the *scelo2; scelo3* + p*ScELO3* control strain (Fig. 2a). The *ZmKCS2* complementing strain had the longest doubling time; fourfold increase as compared to the BY4741 control. The *ZmKCS15* complementing strain had the shortest doubling time, although it was still two- to three-fold longer than the control strains (see Table 1).

**Table 1:** Growth characteristics of the *ZmKCS* complementing strains.

Yeast strain	Doubling time		Time to mid-log phase	Time to stationary phase	Maximum OD600
	h	SE	h	h	
BY4741 + pAG423 (empty)	2.6^F^	0.05	8	15	6.4
BY4742 + pAG423 (empty)	5.0^D^	0.02	15	21	5.7
*scelo2; scelo3* + pScELO3	4.3^E^	0.08	12	18	1.7
*scelo2; scelo3* + *ZmKCS2*	8.3^A^	0.14	18	> 60	1.7
*scelo2; scelo3* + *ZmKCS4*	7.1^B^	0.21	18	> 60	1.6
*scelo2; scelo3* + *ZmKCS11*	7.1^B^	0.11	18	> 60	1.7
*scelo2; scelo3* + *ZmKCS15*	6.4^C^	0.19	12	18	1.7
*scelo2; scelo3* + *ZmKCS20*	7.4^B^	0.02	18	> 60	1.7

Data were collected from strains grown in liquid cultures as described in the “Methods” section. Doubling time is the average from 3 replicate experiments ± standard error (SE), and different superscript letters (^A-F^) indicate statistically significant differences among the strains based upon Tukey HSD (*p* value < 0.05). The ANOVA F-ratio (240.2) and F-value (< 0.0001) were assessed prior to Tukey HSD.

Additionally, the growth of these *ZmKCS* complementing strains were assessed on solid media, grown at either 23 °C or 30 °C (see Supplementary Fig. S5). Similar to the growth observed in liquid media, all the complementing strains grew slower than the BY4741 strain and of these, the *ZmKCS15*-complemented strain was the fastest growing on solid media (see Fig. 2b). At 23 °C, the growth of all the strains were initially delayed, with the greatest impact being on the *ZmKCS4* complement, which showed only minimal growth even after a 168-h period (see Fig. 2b).

**Figure 3:**
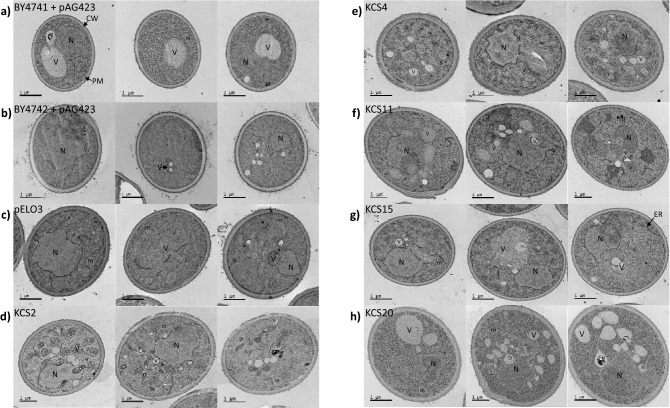
Ultrastructural comparison of *ZmKCS* complementing strains. Transition electron micrographs of three representative yeast cells of each identified genotype. V = vesicle, m = mitochondria, N = nucleus, CW = cell wall, PM = plasma membrane, ER = endoplasmic reticulum. Scale bars = 1 µm.

Figure 3 compares the cellular ultrastructure of the five *ZmKCS* complementing strains to those of the BY4741, BY4742, and *scelo2; scelo3* + p*ScELO3* control strains (see Fig. 3a–c). The most consistent difference between these strains is the increased occurrence of fragmented vacuoles in all the *ZmKCS* complementing strains (see Fig. 3d–h), and the alteration in the thickness of the cell wall. The latter ultrastructural feature was quantifiable, and these data indicate that the thickness of the cell wall is increased by ~ 35% in the *ZmKCS4*-complemented strain, whereas the *ZmKCS15*-complemented strain has a cell wall that is ~ 30% thinner from the control strains (see Supplementary Fig. S4).

**Figure 4:**
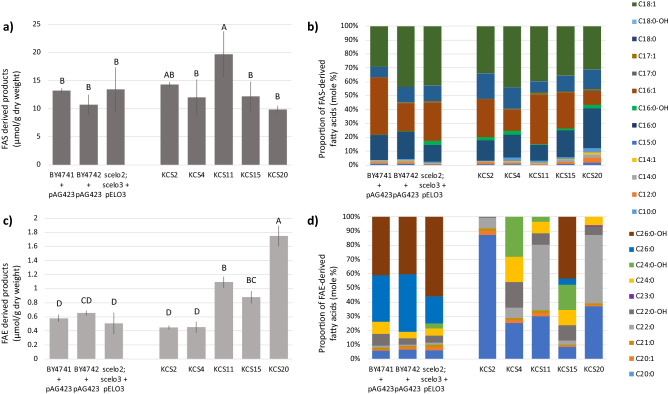
Fatty acid profiles generated by the *scelo2; scelo3* double mutant strains complemented by the expression of *Zm*KCS isozymes. (**a**) Yield of FAS-derived products. (**b**) Proportion of individual FAS-derived products. (**c**) Yield of FAE-derived products. (**d**) Proportion of FAE-derived products. See “Methods” section for details on data gathering and analysis. Fatty acid profiles were determined by GC–FID analysis of FAMEs. Error bars represent standard error from 3 replicates, and different letters above data-bars indicate statistically significant differences based on the Student’s t-test (*p* < 0.05).

**Figure 5:**
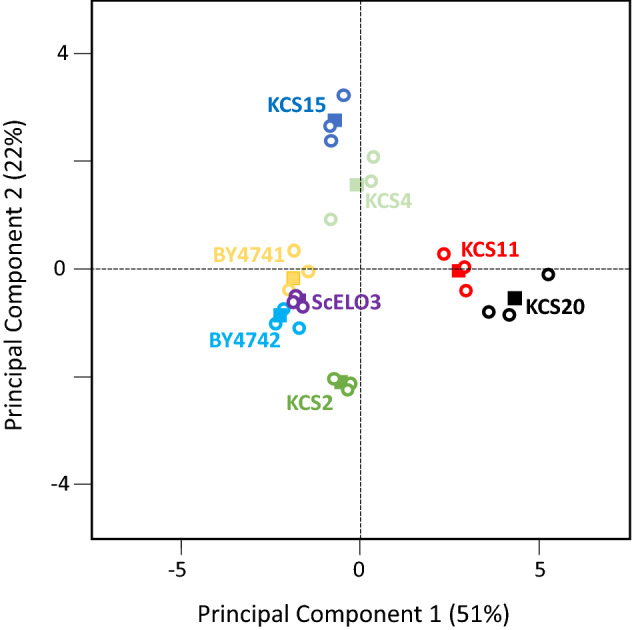
Principal component analysis (PCA) of VLCFA profiles produced by *ZmKCS* complementing strains. These analyses are of data presented in Fig. 4c,d. Open circle data-points are from each of 3 replicate analyses of each genotype, and the average position of these data-points are displayed as the filled data-points.

The fatty acid profiles of the five *ZmKCS*-expressing strains that complement the lethality of the *scelo2; scelo3* double mutant were determined and compared to each other and to the control strains. As may be expected, there are only subtle quantitative or qualitative changes in the fatty acid products synthesized by de novo FAS (i.e., fatty acids of ≤ 18 carbon atoms) (see Fig. 4a,b). However, when one considers the products of the FAE system, there are considerable quantitative and qualitative difference in the VLCFAs produced by these *ZmKCS*-complementing strains (see Fig. 4c,d). Specifically, complementation by *ZmKCS11*, *ZmKCS15*, and *ZmKCS20* results in a ~ two- to four-fold increase in the accumulation of VLCFAs as compared to the control strains (see Fig. 4c). In contrast, whereas both *ZmKCS2* and *ZmKCS4* complement the lethality of the *scelo2; scelo3* double mutant, the resulting strains generate quantities of VLCFAs that resemble the levels seen in the three control strains. Moreover, each of these recovered strains generated VLCFA profiles that are distinct from each other, and markedly different from the profiles of the three control strains (see Fig. 4d). Principal component analysis (PCA) of these data identifies two principal components that together account for ~ 70% of the variation in the data (see Fig. 5). These analyses indicate that whereas the variance in the VLCFAs of the p*ScELO3*-expressing recipient strain correlates to the BY4741 and BY4742 WT strains, all five *ZmKCS* complemented strains present distinct FAE product profiles. Furthermore, these analyses separate the strains based upon their VLCFA profiles (PCA1) or based upon the profiles of the hydroxylated VLCFAs (PCA2). Specifically, the *ZmKCS11* and *ZmKCS20* complementing strains vary from the control strains primarily in the VLCFA profiles, whereas the *ZmKCS2, ZmKCS4* and *ZmKCS15* complementing strains vary from the control strains primarily in the profiles of the hydroxylated VLCFAs.

More specific examination of the FAE generated products identify that whereas the control strains produce predominantly 26-carbon VLCFAs and the 2-hydroxy derivative, only the *ZmKCS15*-complementing strain generates these VLCFAs. The *ZmKCS2* complement predominantly produces 20-carbon fatty acid and cannot elongate fatty acids beyond 22-carbon chain length. The *ZmKCS4* complement accumulates products with chain lengths ranging from 20 to 24-carbons but is unable to elongate beyond this chain length. Both the *ZmKCS11-* and *ZmKCS20*-complementing strains produce abundant quantities of both 20- and 22-carbon products and can only generate smaller quantities of 24-carbon VLCFAs.

## Discussion

Although VLCFAs account for a relatively small portion (< 10%) of the fatty acids that constitute cellular lipids, their significance to biological processes is illustrated by their essentiality to viability. Namely, mutations that block the ability to elongate preexisting fatty acids to VLCFAs present a lethal phenotype in a wide range of phylogenetic clades of eukaryotes^7–9,11,12^. The length of these molecules provides chemophysical characteristics that make them important components of phospholipids, sphingolipids, and glycerolipids within biological membranes. Moreover, in plants these molecules also have a unique role in being precursors to the cuticle, the coating that covers the aerial surfaces of these organisms^3,4,6^. Evolutionarily, the assembly of the cuticle appeared about 450 million years ago and allowed plants to adapt to life on land, a desiccating environment, as compared to the aquatic environment where life is thought to have first arisen^6^.

The isolation and characterization of the enzymatic components of the plant FAE system^8,17,22–24^ has identified a dilemma concerning the significance of the biochemical and genetic redundancy in the enzymes that catalyze some of the reactions of the FAE system. In particular, two distinct enzymes catalyze the Claisen condensation reaction of the FAE cycle, the KCS-type enzymes that occur uniquely in plants, and the ELO-type enzymes that are phylogenetically more widely distributed^17^. Moreover, plant genomes contain a large number of *ELO* genes and even larger number of *KCS* genes^17,18,25–27^. Although this appears to be a characteristic widely dispersed among plant phyla, it is not as prevalent among the other components of the FAE system. Thus, although single copy genes encode the HCD and ECR components in maize and Arabidopsis^17,28,29^, the maize genome carries two paralogs of the KCR component^8^, whereas the Arabidopsis genome encodes only one functional copy of this enzyme^9^. Analysis of each individual maize KCR mutant confers different VLCFA profiles, and it has been suggested that this may be due to different KCR isozymes having different interactions with different fatty acid elongase systems constituted with different KCS and/or ELO isozymes^8,17^.

Addressing this redundancy dilemma, either by in vivo or in vitro strategies, presents several challenges, primarily associated with the integral membrane nature of the FAE system, which makes it difficult to isolate these proteins in their native conformation. Additionally, the number of gene paralogs and the functional redundancy within the FAE system complicate the use of forward or reverse genetics strategies to decipher function. In this study we have used an alternative approach, employing heterologous expression in the yeast *S. cerevisiae*, to characterize the 26 *Zm*KCS paralogs that are identifiable in the maize genome.

Initial characterizations involved the individual expression of the 26 *Zm*KCS paralogs in a yeast strain that carried the endogenous wild-type FAE system; this genetic modification had minor changes on the attributes that we quantified (i.e., cell growth or fatty acid profiles). Subsequent experiments involved the expression of these *ZmKCS* paralogs in recipient mutant strain that did not express the FAE components that catalyze the Claisen-condensation reaction of the yeast system. Yeast carries three genes that encode this catalytic function (*ScELO1*, *ScELO2* and *ScELO3*), and we focused on the latter 2 paralogs because *ScELO1* elongates preexisting fatty acids to only 18-carbon chain length. However, both *ScELO2* and *ScELO3* can produce VLCFAs, with the former being capable of producing 24-carbon fatty acids, and the latter is required for the synthesis of 26-carbon fatty acids^7,30,31^. Moreover, whereas each *scelo2* and *scelo3* single mutants are viable, the *scelo2; scelo3* double mutant exhibits synthetic lethality^7^.

The individual *scelo2* and *scelo3* mutations had opposite quantitative effects in reducing and increasing the accumulation of VLCFAs, respectively. Additionally, only the *scelo3* mutant changed the chain-length profile of the VLCFA products. Yet, the expression of each of the *ZmKCS* proteins in these mutant strains did not substantially alter the fatty acid profiles from those expressed by each single mutant strain. Collectively therefore, these three sets of expression experiments suggest that the *Zm*KCS components are unable to displace the *Sc*ELO components from the *Sc*FAE complex, even when the *Sc*FAE system is disrupted by the absence of one of the three *Sc*ELO proteins that catalyzes the Claisen-condensation reaction required by the native yeast FAE system. This conclusion is consistent with prior studies^31,32^, which found that the yeast FAE system can be co-immunoprecipitated as a partial complex. Moreover, one could speculate that our findings are indicative of a FAE quaternary model in which there are distinct *Sc*ELO2- and *Sc*ELO3-containing FAE complexes, and thus the individual *scelo2* or *scelo3* mutations would not disrupt a mixed *Sc*ELO2/*Sc*ELO3-containing FAE complex, but individually eliminates one of these distinct complexes. Consistent with this supposition, global interactome data of integral membrane proteins in yeast did not provide evidence for *Sc*ELO2 and *Sc*ELO3 interactions^33^, though these interactome data identify interactions of these two proteins with other *Sc*FAE components^31,32^.

This supposition was further explored in a third series of experiments in which we expressed each *Zm*KCS in a *scelo2; scelo3* double mutant strain. Such a strain is synthetically lethal, but its viability was maintained by an ectopic copy of a wild-type *ScELO3* allele. A plasmid-shuffle strategy was used to identify five *ZmKCS* paralogs that are capable of replacing the ectopic *ScELO3* allele and maintaining the viability of the *scelo; scelo3* double mutant. The specific complementing isozymes being *Zm*KCS2, *Zm*KCS4, *Zm*KCS11, *Zm*KCS15 and *Zm*KCS20. These five *Zm*KCS isozymes that can complement the lethality of the *scelo2; scelo3* double mutant belong to two different previously defined phylogenetic KCS-clades, the α/β and the ζ clades^17,25^. Moreover, these *Zm*KCS proteins are inclusive of all members of the ζ clade (i.e., *Zm*KCS2, *Zm*KCS11, and *Zm*KCS20) and all members of the α/β clade (*Zm*KCS4 and *Zm*KCS15). Because primary structure homology defines these clades, one could surmise therefore that the *Zm*KCS proteins in these two clades share common structural feature(s) that facilitates interactions with the other components of the yeast FAE system, and that these structural feature(s) are absent from the other 21 *Zm*KCSs that belong to the other 7 distinct phylogenetic clades, which cannot achieve genetic complementation.

**Figure 6:**
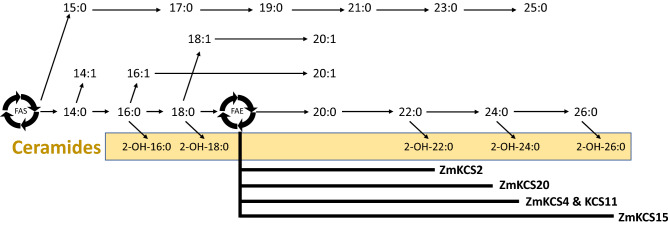
Enzymatic capabilities of the *Zm*KCS isozymes in generating VLCFAs. Metabolic model of the enzymatic capabilities of *Zm*KCS isozymes based on the VLCFA profiles expressed by the *scelo2; scelo3* double mutant strains, complemented by the expression of different *Zm*KCS isozymes. FAS generated fatty acyl-CoAs of up to 18-carbon chain-length can be desaturated to generate monounsaturated fatty acids. FAE elongates these saturated and monounsaturated fatty acids to lengths of up to 26-carbons, and these fatty acids can be hydroxylated at the 2-position after they are incorporated into ceramide lipids. Bold lines at the bottom of the figure identify the differential enzymatic capability of each *Zm*KCS isozyme to catalyze elongation of fatty acids to specific chain lengths.

It’s generally believed that the product profile of the FAE system is a property of the enzyme component that catalyzes the Claisen-condensation reaction of each FAE cycle. Although evidence for this supposition has been gathered for the ELO proteins^7,16,31,34,35^ and KCS proteins^19,26,36–42^, this study, in combination with the prior characterizations of *Zm*KCS4^17^ and *Zm*KCS19^43^, provides a systematic assessment of this hypothesis for the KCS-class of FAE enzymes. The unique VLCFA profiles generated by the five complemented strains reveals differences in the enzymatic capability of each *Zm*KCS (see Fig. 6). We find that the *ZmKCS2* has the ability to elongate fatty acids to 22-carbon, *ZmKCS4, ZmKCS11,* and *ZmKCS20* has the ability to elongate up to 24-carbons, and *ZmKCS15* can elongate up to 26-carbons. One could postulate that the success of the *ZmKCS15* complementation, as manifest by the fastest doubling time of all the recovered strains, may be attributable to the ability of this strain to produce a VLCFA profile that is similar to WT yeast (i.e., the production of 26-carbon fatty acids). Yet this strain still displays impaired growth, as compared to the recipient control strain, and this may be due to alterations in the 2-hydroxy fatty acid profile, which separates the *ZmKCS15* complemented strain from the control strains in the PCA. Indeed, a significant proportion of the recovered VLCFAs are the 2-hydroxy-derivatives, which are associated with the ceramide moiety of sphingolipids. In yeast cells sphingolipids make up 10–20% of the membrane lipids^7^, and these complex lipids serve vital requirements for cellular viability^44^. As with prior studies^20^, we found that in WT yeast the dominant hydroxy-fatty acid incorporated into the ceramide moiety of the sphingolipids is 2-hydroxy-26:0, accounting for over 70% of the fatty acids that are associated with this lipid. The *ZmKCS*-complemented strains express dramatic differences in the accumulation of 2-hydroxy-fatty acid products. Although, the near absence of 2-hydroxy-VLCFAs does not affect the viability of these cells, they do suffer a severe growth penalty. Thus, these strains provide a synthetic biology platform to further explore the dependency between VLCFAs and 2-hydroxy-fatty acid products and the physiology of this organism.

Additionally, the strains we generated in this study are beyond the capabilities of natural evolutionary processes and can provide deeper insights on the substrate specificity and VLCFA-products generation by *Zm*KCS-containing FAE systems. For example, in planta the maize FAE system has the ability to generate VLCFAs of up to 32 and 34 carbons^8,43^. Yet even though KCS appears to be the determinant of the chain length that FAE can generate, the systems we have assembled with *Zm*KCS enzymes do not recapitulate this in planta metabolic capability. There are one or more explanations for this apparent deficiency of the yeast system, including the possibility that this is an attribute associated with the other *Zm*KCS paralogs that did not complement the *scelo2; scelo3* double mutant lethality, or the involvement of *Zm*ELO proteins. Additionally, analysis of plant mutants indicate that the *Zm*KCR components can also affect the FAE product profile^8^. Furthermore, in planta evidence indicates that genetically encoded cofactors (e.g., *Glossy2* and *Glossy2-like*) may enhance the catalytic capability of the terminal elongation cycles of FAE^45^. This latter model is supported by the characterization of the homologous *CER2* gene that appears to modulate the terminal elongation cycles of the Arabidopsis FAE enzyme^4^. These hypotheses can now be directly evaluated by using the yeast platform that we have developed in this study. Specifically, the *ZmKCS-*complementing strains represent a new genetic resource to further explore these questions by individually co-expressing *Zm*KCR isozymes or the *Zm*GL2 coenzymes and directly evaluating how these additional components provide the plant FAE system with the dexterity to generate diverse VLCFA products needed for many metabolic endpoints that include the cuticle, seed oils, and membrane lipids (sphingolipids and phospholipids).

## Methods

### *ZmKCS* expression vectors

Open reading frames (ORFs) of candidate *ZmKCS* sequences have been identified using BLASTP^46^ by sequence homology with maize and Arabidopsis homologs^17^. These ORFs were PCR amplified with appropriate primers from cDNA prepared from RNA isolated from the maize inbred line, B73. RNA was purified from a number of different maize tissues using the Qiagen RNeasy Kit (Hilden, Germany) (see Supplementary Table 1), and RNA was converted to cDNA using SuperScript^™^ Double-Stranded cDNA Synthesis Kit (Invitrogen, Carlsbad, CA). Alternatively, if a *ZmKCS* gene did not contain an intron, the *Zm*KCS ORF was directly amplified from B73 genomic DNA. These PCR reactions were conducted with Phusion high-fidelity DNA polymerase (NEB, Ipswich, MA), and DNA products were isolated after agarose gel electrophoresis using the Qiagen Gel Extraction Kit (Hilden, Germany). For three KCS ORFs, (*ZmKCS9, ZmKCS18* and *ZmKCS19*) sequences were codon optimized for expression in yeast, and chemically synthesized as g-blocks (IDT, Coralville, IA). A ‘CACC’ leader sequence required for entry cloning into pENTR/dTOPO vectors (Invitrogen; Carlsbad, CA) was added to each KCS-coding fragment by PCR amplification or during DNA synthesis. Assembled KCS-carrying pENTR/dTOPO plasmid-vectors were transformed into One-Shot^™^ Mach1^™^ T1 competent *E. coli* cells using a standard protocol (Invitrogen, Waltham, MA). LR clonase II (Invitrogen, Waltham, MA) was used to subclone KCS ORFs into the appropriate Gateway yeast expression vectors, pAG423-GPD and pAG426-GPD^47^. All plasmid-vectors were confirmed by sequencing (Iowa State University DNA Facility, Ames, IA) and *E. coli* strains harboring these plasmids were maintained on Luria–Bertani (LB) media containing the appropriate antibiotics.

### Yeast strains and media

Yeast cultures were grown using standard procedures^48^. Liquid precultures were grown to stationary phase and then diluted into fresh media to an OD_600_ of 0.1 and subsequently grown at 30 °C with constant agitation at 250 rpm for 24 h. The parental strains BY4741 and BY4742 were maintained on YPD (Yeast Peptone Dextrose) media. Yeast strains carrying single gene-deletion mutations were obtained from Open Biosystems (Hunstville, AL) and are listed in Supplementary Table 2. These strains were maintained on YPD + 200 µg/ml Geneticin (G418; Invitrogen, Waltham, MA). The double mutant yeast strain carrying the *scelo2::*KanMX_4_ and *scelo3::*HphMX_6_ disrupted alleles was maintained on YPD + 300 µg/ml Hygromycin (Goldbio, St. Louis, MO). Yeast strains expressing maize *ZmKCS* sequences were selected by their ability to grow on synthetic defined minimal medium (SD) without the appropriate amino acid or nucleobase. Strains that were genetically complemented with *ZmKCS* sequences were maintained on SD-His media containing 1 mg/ml 5-fluoroorotic acid (5-FOA, Goldbio, St. Louis, MO). Where appropriate, a strain that carried an empty expression vector was used as a control.

### Yeast genetics

Plasmid transformation of yeast cells was by a standard lithium acetate protocol^49^. Each of the 26 *ZmKCS* ORFs, cloned in the pAG426-GPD plasmid, were transformed into the BY4741 strain that carried a *scelo2* disrupted mutant allele, and into BY4742 strain that carried a *scelo3* disrupted mutant allele (see Supplementary Table 2). The *ScELO3* gene was cloned with its native promoter sequence into a modified pAG416-GPD (low copy, URA3) plasmid backbone via In-Fusion cloning (Takara Bio USA, Inc., Mountain View, CA). The modification of the pAG416 plasmid removed the GPD promoter from the vector backbone, so that the cloned *ScELO3* gene would be expressed by its native promoter. The resulting plasmid was transformed into a diploid heterozygous strain, carrying *scelo2::*KanMX_4_ and *scelo3::*KanMX_4_ mutant alleles (see Supplementary Table 2). Sporulation of the resulting diploid strain was performed according to Enyenihi and Saunders^50^, and the haploid *scelo2::*KanMX_4_*, scelo3::*KanMX_4_ double mutant, which is normally lethal, was recoverable because it carried the *ScELO3* expressing plasmid that also carried the *URA3* marker. This recovered double mutant was identified based on the inability of the strain to grow on 5-FOA-containing media and was additionally confirmed by PCR genotyping and DNA sequencing of the PCR product. The resistance cassette at the *scelo3::*KanMX_4_ locus was subsequently swapped with HphMX_6_ (pAG32; Euroscarf, Frankfurt, Germany), and this was confirmed by the ability of the strain to grow in the presence of 300 µg/ml hygromycin, and by DNA sequencing of the *scelo3::*HphMX_4_ allele. This strain was used in plasmid shuffle experiments.

### Complementation screen using plasmid shuffle

Plasmid shuffle experiments were performed with the *scelo2*::KanMX_4_, *scelo3*::HphMX_6_ strain that also carries the p*ScELO3(URA3)* plasmid using the method previously described by Truong et al.^51^. In these experiments, the p*ScELO3(URA3)* plasmid was shuffled with pAG423-GPD expression vectors, each expressing one of the 26 *Zm*KCS ORFs. In short, cells from a 600 µl aliquot of a culture grown to an OD600 ~ 6 were pelleted and resuspended in water and spread onto plates containing SD-His media containing 1 mg/ml 5-FOA. Cultures were grown at 30 °C for up to 21 days. To ensure that plates did not dry out during this time, they were placed in a sealed Tupperware container with damp paper towels, which maintained higher humidity. Plates were examined weekly, and as colonies appeared, they were removed, and replica plated for molecular characterizations. These characterizations included PCR confirmation of the loss of the p*ScELO3* (*URA3*) plasmid, and PCR confirmation for the presence of the *ZmKCS* ORF. All PCR products were sequenced for final confirmation.

### Strain growth analysis

Three colonies of each WT, and *scelo2* or *scelo3* mutant yeast strains each expressing individual *Zm*KCS isozymes were grown to stationary phase in liquid precultures. To monitor growth, 100 µl aliquots of these precultures were diluted to an OD600 ~ 0.1 into round-bottom 96 well tissue culture plates (VWR, Radnor, PA). Strains were randomly positioned in these plates and plates were incubated in a BioTek microplate reader (Winooski, VT) for 36 h at 30 °C with constant agitation. Growth was monitored by measuring OD600 every 30 min. Doubling time for each culture was calculated using Gen5 data analysis software (http://www.biotek.com/products/microplate_software/gen5_data_analysis_software.html). Student’s t-tests or Tukey’s Honest Significant Difference tests were applied to statistically evaluate differences in doubling time among strains.

The growth of the *scelo2; scelo3* double mutant strains complemented by the expression of *ZmKCS* constructs were performed manually in liquid cultures, because these strains grew slower than all other strains. Specifically, precultures were diluted to a starting OD600 ~ 0.2 in 250 ml Erlenmeyer flasks containing 50 ml of SD media supplemented with 1 mg/ml 5-FOA and the necessary amino acids or nucleobases. Cultures were grown with constant agitation at 200 rpm at 30 °C. Three aliquots were collected from each culture and the OD600 of the cultures were monitored until they reached stationary phase of growth. Growth of these complementing strains was also monitored on solid SD media supplemented with 1 mg/ml 5-FOA and the necessary amino acids. In these experiments, a liquid preculture of each strain was diluted to an OD600 ~ 0.3, and serially diluted tenfold, and triplicate aliquots of each dilution were spotted on the solid media. Positions for each genotype were randomized on the plates. Plates were wrapped with Parafilm to reduce evaporation and incubated at either 23 or 30 °C in a sealed plastic Tupperware container with a paper towel dampened with sterile water. At 24-h intervals these plates were imaged using a Fotodyne (Hartland, WI) equipped with a Grayscale Digital Camera (Scion Corporation, Frederick, MD), using the software Foto/Analyst Investigator Eclipse PC-Firewire.

### Analysis of fatty acids

Fatty acids were extracted from an accurately weighed lyophilized cell pellet-aliquot, of between 5 and 15 mg each. Prior to extraction, the cell pellet was spiked with a known amount of nonadecanoic acid internal standard (Sigma Aldrich, St. Louis, MO), and the mixture was pulverized with glass beads (425–600 µm; Sigma Aldrich, St. Louis, MO). Fatty acid methyl esters (FAMEs) were produced by the addition of 1 mL of 5% sulfuric acid in methanol and incubating at 85 °C for 80 min. After cooling, 2 mL of 0.9% (w/v) NaCl was added, and FAMEs were recovered by extracting with two aliquots of 4:1 hexane:chloroform, which were pooled and concentrated by evaporation. Samples were then derivatized with N,O-Bis(trimethylsilyl)trifluoroacetamide with 1% trimethylchlorosilane (BSTFA-TMSC, Sigma Aldrich, St. Louis, MO) and analyzed with either GC–MS or GC–FID^17^. The Agilent software ChemStation was used for peak alignment of GC–FID data along with a parallel GC–MS analysis to chemically identify peaks. Chromatograms and mass-spectral data were queried against an in-house mass spectral library and the NIST 14 Mass Spectral Library using the NIST AMDIS software.

### Transition electron microscopy (TEM)

Cells for TEM imaging were collected from yeast cultures that were grown to mid-log phase. An aliquot of such a culture was mixed with an equal volume of fixative (2% (w/v) paraformaldehyde and 6% (w/v) glutaraldehyde in 0.2 M sodium cacodylate buffer), and the mixture was incubated at room temperature for 5 min. Fixed cells were collected by centrifugation at 1500 × g for 5 min, and the supernatant was removed. The cell pellet was resuspended in 1% (w/v) paraformaldehyde and 3% (w/v) glutaraldehyde in 0.1 M sodium cacodylate buffer and incubated overnight at 4 °C. Each subsequent step included re-suspension and pelleting of cells at 1500 × g for 5 min prior to solution changes. Samples were washed 2 times with 0.1 M cacodylate buffer for 10 min each, 2 times with distilled water for 10 min each, and then post-fixed with 1% (w/v) aqueous potassium permanganate for 1 h at room temperature. Samples were then washed 3 times with deionized water for 15 min each, and *en bloc* stained for 1 h using 2% (w/v) aqueous uranyl acetate. Samples were then washed with distilled water for 10 min, and dehydrated through a graded ethanol series (25, 50, 70, 85, 95, 100% (v/v)) for 1 h for each step. Samples were further dehydrated with three 15-min changes of acetone, and infiltrated with EmBed 812 formula (hard) for EPON epoxy resin (Electron Microscopy Sciences, Hatfield, PA). This final infiltration involved 6–12 h incubations in graded resin:acetone mixtures (1:3, 1:1, 3:1), and final incubation in pure epoxy resin. Cells suspended in the resin were placed into beem capsules and the resin was polymerized at 70 °C for 48 h. Fifty-nm thick microscopic sections were prepared using a Leica UC6 ultramicrotome (Leica Microsystems, Buffalo Grove, IL), and these were collected onto single slot carbon film grids. Grids were post stained for 30 min with 2% (w/v) aqueous uranyl acetate, followed with a 30-min treatment with Reynold’s lead stain to enhance contrast. TEM images were collected using a 200 kV JEOL JSM 2100 scanning transmission electron microscope (Japan Electron Optics Laboratories, USA, Peabody, MA), equipped with a GATAN One View 4K camera (Gatan inc., Pleasanton, CA). Cell wall thickness was measured using ImageJ 1.53a (https://imagej.nih.gov/ij/).

### Data analysis and statistical methods

Data are presented as mean values with standard error. Comparisons with a *p* value < 0.05 were considered statistically significant. Data processing was performed in Microsoft Excel (2021). Statistical tests were carried out using JMP Pro 16 (SAS Institute Inc.; Cary, NC). Data were evaluated for statistical rigor based upon a 2-sided unpaired *t* test or ANOVA followed by post-hoc Tukey’s Honest Significant Difference test (see Supplementary Table S3). Principal component analysis (PCA) was performed using JMP Pro 16. Images were assembled with Microsoft PowerPoint (2021), without any manipulation of image quality parameters.

## Supplementary Information

Supplementary Information 1.

Supplementary Information 2.

## Data Availability

Stenback, KE., Flyckt, KS., Hoang, T., Campbell AC., & Nikolau, BJ. Fatty acid profiles of yeast strains individually expressing 26 *Zea mays* KCS isozymes. Iowa State University public data repository: https://doi.org/10.25380/iastate.17131460.v1. Stenback, KE., Flyckt, KS., Hoang, T., Campbell AC., & Nikolau, BJ. Growth data of yeast strains individually expressing 26 *Zea mays* KCS isozymes. Iowa State University public data repository: https://doi.org/10.25380/iastate.17131460.v1. Stenback, KE., Flyckt, KS., Hoang, T., Campbell AC., & Nikolau, BJ. Cell wall thickness of yeast mutant strains genetically complemented by the expression of *Zea mays* KCS isozymes. Iowa State University public data repository: https://doi.org/10.25380/iastate.17131460.v1.
