# Supplementary material for: Modifying the yeast very long chain fatty acid biosynthetic machinery by the expression of plant 3-ketoacyl CoA synthase isozymes
Source: Sci Rep. 2022 Aug 2;12:13235. doi: 10.1038/s41598-022-17080-8 (PMC9346008; doi:10.1038/s41598-022-17080-8)
Supplement: Supplementary file 1 — Supplementary Information 1. [file 41598_2022_17080_MOESM1_ESM.docx]

# Modifying the yeast very long chain fatty acid biosynthetic machinery by the expression of plant 3-ketoacyl CoA synthase isozymes

Kenna E. Stenback^1,#a^, Kayla S. Flyckt^1,#b^, Trang Hoang^1,#c^, Alexis A. Campbell^1,#d^, and Basil J. Nikolau^1,2*^

^1^Roy J Carver Department of Biochemistry, Biophysics and Molecular Biology, Iowa State University, Ames, Iowa, USA

^2^Center for Metabolic Biology, Iowa State University, Ames, Iowa, USA

^#a^Current Address: Department of Biological Chemistry and Molecular Pharmacology, Harvard

Medical School Blavatnik Institute, Boston, Massachusetts, USA

^#b^Current Address: Corteva Agriscience, Johnston, Iowa, USA

^#c^Current Address: Department of Chemical Engineering, University of Michigan, Ann Arbor, Michigan, USA

^#d^Current Address: School of Education, Iowa State University, Ames, Iowa, USA

^*^Corresponding Author

**Table S1: Sequences of DNA primers used to PCR amplify ORFs of individual *Zm*KCS proteins.**

| **Gene ID** | **KCS identifier** | **Primer Sequence (5' to 3')** |
| --- | --- | --- |
| **Zm00001d030343** | **1** | CACCATGGAGTCCGCGCCGTCA |
|  |  | TCAGATGTACTCTTTTGGCGGG |
| **Zm00001d039094** | **2** | CACCATGGCCGACGACGACCCAAG |
|  |  | TCAGATGGCTGACACCTTGGGG |
| **Zm00001d018455** | **3** | CACCATGGAGCTGCTCGCCCTG |
|  |  | TACGAAGCCGCTCCCCTG |
| **Zm00001d009608** | **4** | CACCATGGACGGAGTCTCCGCC |
|  |  | CTATTGCTGCGTGGGGAAG |
| **Zm00014a017507** | **5** | CACCATGCCGACCGGCGGCGT |
|  |  | TCAGAGGCTGACGACCTCCGGC |
| **Zm00001d028241** | **6** | CACCATGCCGAGCGGCGGCGT |
|  |  | TCAAAGCTTGACGACCTCCGGG |
| **Zm00014a004344** | **7** | CACCATGGCGCCGGGATCGTC |
|  |  | TCAATCAAGGGCCTCCATC |
| **Zm00001d032948** | **8** | CACCATGGCGGTGATGAGCCG |
|  |  | TTAACCAAGAGAGATCTTGTACGTG |
| **Zm00001d045660** | **9** | CACCATGGCTGTGTATAGG |
|  |  | TCAGTGTTTTAAGACATCTGG |
| **Zm00001d027904** | **10** | CACCATGGCGCGGGAGGAGCAG |
|  |  | CTAGGCGTCCATGCGCGC |
| **Zm00001d016438** | **11** | CACCATGGAAAACCCGGCGCC |
|  |  | CAATGCATTTCAGTCGCTTGAA |
| **Zm00001d020206** | **12** | CACCATGCAGAGCTCGGTGG |
|  |  | TCAAGCATGCTTGTGCGTC |
| **Zm00001d047931** | **13** | CACCATGGAGGAGGCTGCGCCC |
|  |  | TCAGACGTACGCCTTGGGAG |
| **Zm00001d028406** | **14** | CACCATGGAGTCTGCTCCCGCC |
|  |  | TCAGACGTACGCCTTGGGA |
| **Zm00001d039053** | **15** | CACCATGAACGGAGGCGCCGC |
|  |  | CTATTGGTGTGCGGGGAAGCC |
| **Zm00001d014168** | **16** | CACCATGGTGGGTAGCAGGGAG |
|  |  | TCAGGCATGGGAGACAGAG |
| **Zm00001d037328** | **17** | CACCATGGGTTCGTCGGCGGC |
|  |  | GGATAGCGGCTAGTGCTTGAGC |
| **Zm00001d027766** | **18** | CACCATGGCTTCTCTTAACC |
|  |  | TTAAGCGTGTTGCGCATTC |
| **Zm00001d032728** | **19** | CACCATGGAACTTGTAACTATG |
|  |  | TCATAAGTCGATCAAATCC |
| **Zm00001d046444** | **20** | CACCATGGAGACGTCAGCGCCG |
|  |  | TCAAGCGCCACCAACCTTTG |
| **Zm00001d009354** | **21** | CACCATGAACACGCTACTGCAT |
|  |  | ATGCCCTAGGAGTCATCAGAT |
| **Zm00001d044579** | **22** | CACCATGGCCAAGCTTCTCAAGC |
|  |  | TCAGATGTTGTCCTTGGCG |
| **Zm00001d051787** | **23** | CACCATGGGCTCCTCGGCGCA |
|  |  | TCAGTGCTTGAGCACGTCG |
| **Zm00001d029350** | **24** | CACCATGCAAGTAATATATCACGCATTC |
|  |  | TTAGAACATGAGGGTGTCGC |
| **Zm00014a030515** | **25** | CACCATGGAGCTACTTCCTCTGC |
|  |  | TTAGAGCATCAGGGTATCGC |
| **Zm00001d021200** | **27** | CACCATGGACATAGCTCACCGAGAC |
|  |  | TTAGGTATCTGTCACAGGGTAC |

**Table S2: Yeast strains used in this study.**

| **Yeast Strains** |  |  |  |  |
| --- | --- | --- | --- | --- |
| **Strain** | **Gene ID** | **Description** | **Relevant Genotype** | **Source** |
| BY4741 (WT) |  | BY4741; Mat a Wild Type | BY4741: Mat a; his3D1; leu2D0; met15D0; ura3D0 | Open Biosystems |
| pAG423 (empty)/BY4741 (WT) |  | Empty vector pAG423 in BY4741 | BY4741: Mat a; his3D1; leu2D0; met15D0; ura3D0 | This Work |
| BY4742 (WT) |  | BY4742; Mat α Wild Type | BY4742: Mat α; his3D1; leu2D0; lys2D0; ura3D0 | Open Biosystems |
| pAG423 (empty)/BY4742 (WT) |  | Empty vector pAG423 in BY4742 | BY4742: Mat α; his3D1; leu2D0; lys2D0; ura3D0 | This Work |
| *scelo2* | ELO2, FEN1, YCR034w | Mat a haploid knockout strain | BY4741: Mat a; his3D1; leu2D0; met15D0; ura3D0; YCR034w::kanMX4 | Open Biosystems |
| *scelo3* | ELO3, SUR4, YLR372w | Mat a haploid knockout strain | BY4741: Mat a; his3D1; leu2D0; met15D0; ura3D0; YLR372w::kanMX4 | Open Biosystems |
| *scelo2; scelo3* | YCR034w, YLR372w | Heterozygous diploid knockout strain | BY4743: Mat a/alpha: his3D1/ his3D1; leu2D0/ leu2D0; lys2D0/LYS2; MET15/met15D0; ura3D0/ ura3D0; YCR034w::kanMX4; YLR372w::kanMX4 | Campbell et al., 2019 |
| *pScELO3/scelo2; scelo3* | YCR034w, YLR372w | Heterozygous diploid knockout strain sporulated to obtain haploid double knockout strain maintained by p*ScELO3* (pAG413, low-copy, URA3, *PELO3-ELO3* | Sporulated BY4743; his3D1; leu2D0; met15D0; ura3D0::URA3/ZmKCS; YCR034w::kanMX4; YLR372w::hphMX4 | This Work |
| *ZmKCS2*/sc*elo2; scelo3* | Zm00001d039094 | *ZmKCS2* complementing the *scelo2: scelo3* double knockout | Sporulated BY4743; his3D1; leu2D0; met15D0; ura3D0::URA3/ZmKCS; YCR034w::kanMX4; YLR372w::hphMX4 | This Work |
| *ZmKCS4*/sc*elo2; scelo3* | Zm00001d009608 | *ZmKCS4* complementing the *scelo2: scelo3* double knockout | Sporulated BY4743; his3D1; leu2D0; met15D0; ura3D0::URA3/ZmKCS; YCR034w::kanMX4; YLR372w::hphMX4 | This Work |
| *ZmKCS11*/sc*elo2; scelo3* | Zm00001d016438 | *ZmKCS11* complementing the *scelo2: scelo3* double knockout | Sporulated BY4743; his3D1; leu2D0; met15D0; ura3D0::URA3/ZmKCS; YCR034w::kanMX4; YLR372w::hphMX4 | This Work |
| *ZmKCS15*/sc*elo2; scelo3* | Zm00001d039053 | *ZmKCS15* complementing the *scelo2: scelo3* double knockout | Sporulated BY4743; his3D1; leu2D0; met15D0; ura3D0::URA3/ZmKCS; YCR034w::kanMX4; YLR372w::hphMX4 | This Work |
| *ZmKCS20*/sc*elo2; scelo3* | Zm00001d046444 | *ZmKCS20* complementing the *scelo2: scelo3* double knockout | Sporulated BY4743; his3D1; leu2D0; met15D0; ura3D0::URA3/ZmKCS; YCR034w::kanMX4; YLR372w::hphMX4 | This Work |

**Table S3: Statistical analyses of *ZmKCS* expressing and *ZmKCS* complementing yeast strains.** ANOVA F-ratio and F-values were determined followed by post-hoc Tukey HSD. Different letters indicate statistical difference based upon Tukey HSD (p-value<0.05) for individual metabolites based upon analysis of three replicates.

Excel file: “Table S3”

**Fig. S1. Growth rates and fatty acid profiles of WT yeast strains expressing each ZmKCS isozyme.** A) Culture doubling time. B) Yield of FAS-derived products. C) Proportion of individual FAS-derived products. D) Yield of FAE-derived products. E) Proportion of individual FAE-derived products. See Methods for details on data gathering and analysis. Fatty acid profiles were determined by GC-MS analysis of FAMEs. Error bars represent standard error from 3 replicates, and “*” identifies statistical significance from the recipient WT strain, based on Student’s t-test (p<0.05).

**Fig. S2. Growth rates and fatty acid profiles of *scelo2* yeast strains expressing *ZmKCS* isozyme** A) Culture doubling time. B) Yield of FAS-derived products. C) Proportion of individual FAS-derived products. D) Yield of FAE-derived products. E) Proportion of FAE-derived products. See Methods for details on data gathering and analysis. Fatty acid profiles were determined by GC-MS analysis of FAMEs. Error bars represent standard error from 3 replicates, and “*” identifies statistical significance from the recipient *scelo2* strain, based on Student’s t-test (p<0.05).

**Fig. S3. Growth rates and fatty acid profiling of *scelo3* yeast strains expressing *ZmKCS* sequence**. A) Culture doubling time. B) Yield of FAS-derived products. C) Proportion of individual FAS-derived products. D) Yield of FAE-derived products. E) Proportion of FAE-derived products. See Methods for details on data gathering and analysis. Fatty acid profiles were determined by GC-MS analysis of FAMEs. Error bars represent standard error from 3 replicates, and “*” identifies statistical significance from the recipient *scelo3* strain, based on Student’s t-test (p<0.05).

**Fig. S4. Cell wall thickness of *ZmKCS* complementing strains.** The cell wall thickness of three representative cells of each strain was measured at three locations around the perimeter of the cells. Average values, with the indicated standard error, were evaluated by Tukey HSD (p-value<0.05), and different letters above each data-bar indicate statistical difference among the strains.

**Fig. S5. Growth of *ZmKCS* complementing strains on solid media.** Serial 10-fold dilution inoculum of each indicated strain was applied on solid media, and growth was evaluated at 23 °C and 30°C at the indicated times after inoculation. Images are representative from three repetitions of this experiment.
